# Metabolic Regulation of Longevity by One-Carbon Metabolism and Flavin-Containing Monooxygenase

**DOI:** 10.1093/geroni/igaa057.414

**Published:** 2020-12-16

**Authors:** Christopher Choi, Scott Leiser, Charles Evans, Daniel Beard

**Affiliations:** 1 University of Michigan Medical School, Ann Arbor, Michigan, United States; 2 University of Michigan, Ann Arbor, Michigan, United States

## Abstract

Nematode flavin-containing monooxygenase-2 (fmo-2) is induced by dietary restriction and hypoxia, and is required for the longevity and health benefits of these pathways. It is also sufficient to confer these benefits when overexpressed. As FMOs are well-conserved across taxa, the fmo-2 mechanism has high translational potential. To determine the changes that occur following fmo-2 induction, we performed RNA-seq and untargeted metabolomics analyses. Our data reveal that one-carbon metabolism (OCM) is significantly altered by fmo-2 overexpression. OCM is a nexus for essential metabolic pathways, including transmethylation, transsulfuration, nucleotide synthesis, and amino acid metabolism. We hypothesized that fmo-2 confers longevity benefits by altering key metabolic processes within or downstream of OCM. To test this, we asked whether fmo-2 and OCM interact to regulate longevity by knocking down expression of genes involved with OCM and measuring lifespan and oxidative stress resistance. To understand the biological implications of these interactions, we generated a computational model using qPCR data of key OCM-related genes to predict changes in OCM metabolic flux. Our model predicts significant changes in OCM flux that are regulated by fmo-2 expression levels and are consistent with our RNAi and multi-omics results. We are now testing this model by knocking down genes downstream of OCM to determine their role in fmo-2-mediated benefits. Preliminary results support our hypothesis that changes in metabolic flux through OCM are involved downstream of fmo-2 expression, and may also implicate the UPRER in this pathway. Our future work will elucidate this mechanism and link stress perception and fmo-2-mediated longevity.

